# Automatic social comparison: Cognitive load facilitates an increase in negative thought accessibility after thin ideal exposure among women

**DOI:** 10.1371/journal.pone.0193200

**Published:** 2018-03-28

**Authors:** Yvana Bocage-Barthélémy, Armand Chatard, Nematollah Jaafari, Nina Tello, Joël Billieux, Emmanuel Daveau, Leila Selimbegović

**Affiliations:** 1 Université de Poitiers, Université de Tours, and CNRS, Poitiers, France; 2 Service Hospitalo-Universitaire de Psychiatrie et de Psychologie Médicale, Centre Hospitalier Henri Laborit, Poitiers, France; 3 Université du Luxembourg, Esch-sur-Alzette, Luxembourg; 4 Université catholique de Louvain, Louvain-La-Neuve, Belgique; 5 University of Reading, Reading, United Kingdom; Technion Israel Institute of Technology, ISRAEL

## Abstract

Women are routinely exposed to images of extremely slim female bodies (the thin ideal) in advertisements, even if they do not necessarily pay much attention to these images. We hypothesized that paradoxically, it is precisely in such conditions of low attention that the impact of the social comparison with the thin ideal might be the most pronounced. To test this prediction, one hundred and seventy-three young female participants were exposed to images of the thin ideal or of women’s fashion accessories. They were allocated to either a condition of high (memorizing 10 digits) or low cognitive load (memorizing 4 digits). The main dependent measure was implicit: mean recognition latency of negative words, relative to neutral words, as assessed by a lexical decision task. The results showed that thin-ideal exposure did not affect negative word accessibility under low cognitive load but that it increased it under high cognitive load. These findings are consistent with the hypothesis that social comparison with the thin ideal is an automatic process, and contribute to explain why some strategies to prevent negative effects of thin-ideal exposure are inefficient.

## Introduction

Abundant research and several meta-analyses have documented the adverse effects of thin-ideal exposure on women’s psychological well-being [1–3]. When confronted with images of the thin ideal in the media, most women must cope with the fact that their body shape fails to match the standards. This failure to meet the cultural standard of beauty usually results in adverse effects, such as negative affect [4], dissatisfaction with one’s own body shape [5], or a temporary increase negative thoughts accessibility [6]. To date, social comparison theory provides the most common and consensual explanation of these effects [7]. However, the question of the automaticity of social comparison with the thin ideal—and, more generally, the question of the automaticity of social comparison—remains open. This is an important question for at least three reasons. First, when women are exposed to the thin ideal in everyday life, they do not necessarily allocate much attention to such images. They may be driving by a billboard featuring the thin ideal, they may be engaged in other daily activities when the thin ideal is featured on the TV screen, or they may be reading an article in a magazine that features advertisement with thin ideal pictures on adjacent pages [8]. Therefore, given the negative consequences that exposure to the thin ideal can produce, it is important to know whether these consequences can occur even if the images are not attended to, but are nevertheless seen. Determining if social comparison is automatic is also important because strategies deployed to attenuate or suppress the negative consequences of social comparison will be tailored differently as a function of whether this process is controlled or automatic. If it is controlled, then it is possible to educate people about social comparison, to underline the relevance/irrelevance of certain standards, or their unattainable nature, in order to avoid the generally negative consequences of upward comparison. However, if the process is automatic, then such explicit strategies might (and do) fail [9–14]. In that case, as researchers we should look into the possibilities of developing other strategies, based on more implicit processes. Finally, from a more fundamental viewpoint, knowing whether a cognitive process is controlled or automatic is valuable in itself, even if there were no immediate practical implications. Accordingly, the aim of the present research is to test the prediction that adverse effects of thin-ideal exposure can be observed even when participants are experiencing high cognitive load. Such a finding would indeed further support the automatic nature of social comparison effects and the automaticity of social comparison with the thin ideal.

Past research has identified social comparison as a process that plays an important role in the occurrence of negative effects of thin ideal exposure. Social comparison has thus been documented as an important *moderator* of adverse effects of thin-ideal exposure [3]. For instance, when women have specific instructions to compare one’s physical appearance with media images, adverse outcomes of thin ideal exposure are facilitated [15,16]. In addition, Bessenoff [17] showed that social comparison also *mediates* the impact of thin-ideal exposure on diverse negative psychological consequences (depression, dysphoric mood, diminished self-esteem, and weight concerns). Finally, a meta-analysis by Myers and Crowther [18] supported the link between upward appearance comparison and body dissatisfaction. Taken together, this body of research strongly supports the idea that social comparison of appearance underlies the negative effects of thin-ideal exposure. However, to date, it is unclear whether the social comparison is an automatic or a controlled process. In order to tailor adapted strategies to prevent negative effects of thin-ideal exposure, it is crucial to identify the nature of the processes involved. In the next section, we review previous research relevant to the question of the automaticity of social comparison. We first focus on awareness, controllability, and intentionality features, which are indirectly related to our specific research question. Then, we discuss prior work related to the question of whether social comparison can occur under cognitive load, with special attention devoted to studies that have relied on the thin ideal as the target of comparison. We underline the inconclusive nature of the relevant findings and attempt to bring a contribution to this line of research with the present study.

### Automaticity of social comparison to the thin ideal

A direct approach to test the automaticity of a specific process is to test whether it respects the four criteria of automaticity defined by Bargh [19]: lack of awareness, lack of control, lack of intentionality, and efficiency (resource-independence). Some previous work unrelated to the thin ideal has suggested that social comparison may indeed be a process that can run outside of awareness [20]. With reference to body image and media concerns, Jansen and deVries [21] exposed Dutch women to subliminal thin-ideal pictures and found no evidence that this exposure affected participants’ self-esteem, mood, or eating behaviors. Thus, these findings do not support the hypothesis of automatic social comparisons, at least in relation to the *awareness* criterion. It is worth noting that these studies are to some extent underpowered. The sample sizes ranged from 52 to 59 for 3 or 4 independent groups of participants. In contrast, Chatard, Bocage-Barthélémy, Selimbegovic and Guimond [22] recently conducted two high-powered experiments in which young women were subliminally exposed (or not) to thin-ideal pictures. The results demonstrated that women reported greater body appearance anxiety following subliminal exposures to the thin-ideal body compared with a no-models control condition. Thus, social comparison can occur outside of awareness and is automatic with regard to this specific criterion.

To our knowledge, the feature of *controllability* has not yet directly been tested in relation to social comparison [23]. In addition, only one study appears to suggest that social comparison could be an *unintentional* process [24]. In this diary study, 51.8% of participants declared that they compared their appearance to another person’s unintentionally. However, if social comparison can occur outside of awareness, then it is likely that for a number of spontaneous comparisons individuals are even unable to report on the unintentionality of the process. Indeed, as underlined by Bargh [19], when individuals are unaware of a stimulus, they cannot control their reactions to that stimulus or regulate these reactions intentionally. In other words, studies that use subliminal presentations of stimuli are relevant not only to the awareness criterion of automaticity but also to the control and intention criteria. Consequently, the two most frequently used techniques to test automaticity are the subliminal presentation of stimuli and cognitive load manipulations.

Taken together, research conducted on *awareness*, *controllability*, and *intentionality* is fairly limited and does not provide a clear account of the automaticity of the social comparison process. In addition, as automaticity features may sometimes occur separately (i.e., a process may have some, but not all features of automaticity), these studies are not informative as to whether social comparison is resource-independent, which is the focus of the present study.

In relation to this, research on the *efficiency* of social comparison is also relatively scarce. Gilbert, Giesler, and Morris [25] argued that social comparison is resource-independent based on data suggesting that cognitive load does not impair comparison processes even when the target of comparison is clearly irrelevant. In relation to thin-ideal exposure, Brown and Dittmar’s [26] findings suggested that social comparison may be an efficient process, at least for women who strongly internalize the thin ideal as a personal standard of beauty. Women were exposed to thin-ideal pictures for either 150 ms or 10 seconds or were not exposed at all in the control condition. The authors argued that 150 ms of exposure, although not subliminal, does not allow an in-depth processing of the images, while 10 s of exposure does. Consistent with this idea, women exposed to the thin ideal for 10 seconds reported more weight-related anxiety than women exposed for just 150 ms and those who were not exposed at all. In addition, there was a main effect of thin-ideal internalization such that high levels of internalization facilitated body-related anxiety upon exposure. Importantly, exposure length did not qualify this effect, suggesting that independently of the depth of processing (as operationalized by exposure length), high internalizers appeared to engage in social comparison with the models. In other words, women with specific characteristics appeared to engage in a social comparison process quite effortlessly and efficiently. Unfortunately, both of these studies were also low-powered, having either 68 participants for 4 independent groups [25] or 75 participants for 3 independent groups [26]. In fact, power analyses suggest that to have 80% power to detect a medium-sized effect (Cohen’s d = 0.50) in an ANOVA with 3 or 4 independent groups, the sample has to have at least 128 participants. These findings are thus to be taken into account with some caution and demand replication. However, Brown and Dittmar’s findings suggest thin-ideal internalization as a construct that might facilitate automatic social comparison with the thin ideal. Obviously, some women tend to internalize the thin ideal as a standard of comparison more than others, at least at the explicit level. However, it is likely that all women internalize the thin ideal to some extent, through socialization processes and the related influence of family, peers, and the media [27]. In the media, the thin ideal is ubiquitous and glorified as a standard of female beauty, while body dissatisfaction, desire for thinness, and “fat talk” (negative conversations about one’s body shape) seem to be normative among young women [28]. Indeed, some studies suggest that in addition to being a factor of vulnerability to thin ideal exposure effects, thin ideal internalization also increases after exposure to the thin ideal (as incarnated by the Barbie doll[29]). Taken together, these studies suggest that there might be a bidirectional causal relation between thin ideal exposure and thin ideal internalization. In fashion magazines and some advertisements, women are often explicitly encouraged to compare their body shape to that of the models (e.g., “Are you beach-body ready?”) with a thin ideal picture [30]. As suggested by a large number of studies, the repetition of a process leads that process to become automatic and thus independent from resource availability [31]. Thus, repeated comparison between the self and the standard of thinness as well as the normative thin ideal internalization both foster the automaticity of the social comparison to the thin ideal. However, to the best of our knowledge, there is no conclusive evidence for the efficiency (i.e., resource-independence) feature of social comparison [22].

In contrast to the research described above, recent work having investigated whether social comparisons with media are efficient, i.e. require few cognitive resources, suggested that social comparisons are not efficient mental processes [23]. In Study 1, women had to retain either 8 different digits (high cognitive load condition) or one digit repeated eight times (low cognitive load condition) during thin-ideal exposure. Visual analogue scales were used to assess the self-evaluation of physical appearance and the level of negative affect pre- and post-exposure. While women in the low load condition suffered an increase in negative mood and a decrease in self-evaluated appearance after thin-ideal exposure, no changes were found among women in the high load condition. In Study 2, Want et al. [23] examined whether evidence of efficiency in social comparisons could be found among a subset of participants. The procedure was practically identical as the one in Study 1, with the exception that a scale to determine to what extent participants internalized media images as important sources of information regarding appearance was added (The Sociocultural Attitudes Towards Appearance Questionnaire (SATAQ-3), [32]). This scale allowed evaluating the extent to which women feel pressured to emulate the appearance of models, movie stars, and TV celebrities. Women characterized by high scores on the SATAQ tend to compare themselves more frequently with media images than those with lower scores. These results suggested that participants under condition of high cognitive load were not affected by exposure to the images even if they scored high on the SATAQ. In sum, Want et al. [23] found no evidence supporting that social comparisons are efficient mental processes. In a second study, Want & Saiphoo [33] replicated the procedures and analyses from Want et al. [23] but used a selected sample of women who reported that they habitually compared themselves with media images. They found the same pattern of results: women in the cognitively busy condition and in the control condition (without image exposure) did not experience an increase in negative mood or a decrease in appearance satisfaction. However, women under low cognitive load who had been exposed to the thin ideal showed increased negative mood and decreased appearance satisfaction from before to after exposure. As these studies were appropriately powered (with 58 and 59 participants per group for Studies 1 and 2, respectively), they provide the most reliable results pertaining to our research question. Want et al. [23] and Want and Saiphoo [33] used explicit measures (self-reported mood and self-reported appearance satisfaction). As argued by Want et al. [23], one limitation to their research is the explicit nature of the outcome measure. They underlined that further research remains necessary to examine whether cognitive load affects reactions to thin-ideal exposure assessed with implicit dependent measures. Indeed, responses on explicit, self-reported measures are controllable: people report what they are aware of and/or what they are willing to report [34]. Another limitation of explicit measures is the demand characteristics. Mills, Polivy, Herman and Tiggemann [35] examined whether demand characteristics would result in worsened mood following exposure to ideal images. They found that, following thin-ideal exposure, the presence of explicit demand characteristics led women to report worse feelings than the presence of minimized demand characteristics. Their findings provided evidence that explicit measures are susceptible to biases, due to social desirability and demand characteristics. This is even truer in repeated-measures designs, in which the same explicit measure is completed twice by participants, as is the case in the studies of Want and his collaborators. Therefore, the question of automaticity of social comparison to the thin ideal warrants further study, especially in relation to implicitly measured outcomes.

### Study overview

The present study aims to examine the effect of cognitive load on reactions to thin-ideal exposure, as manifested on an implicit dependent measure. Female participants were exposed to images of the thin ideal or women’s fashion accessories. A classic implicit measure, i.e. a lexical decision task, was used to assess the accessibility of negative concepts following thin-ideal exposure. Half of the participants were allocated to a low cognitive load condition and the other half under high cognitive load condition. According to Payne [36], a process can be considered automatic if it occurs as strongly, or more strongly, under cognitive load as it does in the absence of load. Following this reasoning, the data would be consistent with our hypothesis if we found a main effect of thin-ideal exposure such that it increased negative thought accessibility regardless of cognitive load, or if we found an interaction such that thin-ideal exposure increased negative thought accessibility to a higher degree under high than low cognitive load. In contrast, the data would be inconsistent with our hypothesis if thin-ideal exposure increased negative thought accessibility more under low load than under high load [23, 33].

Importantly, in contrast to much of the previous research (with the notable exception of Want et al.’s studies), we ensured high statistical power to detect the interaction of cognitive load and thin-ideal exposure, relevant to our hypothesis.

## Method

### Power analysis

An analysis was conducted to estimate the sample size necessary to achieve the recommended level of 80% power to detect a medium-sized effect (Cohen’s d = 0.50) in an ANCOVA with one covariate and 4 independent groups [37]. To do this, we relied on G*Power software [38]. The analysis yielded a sample size of 128. Thus, we decided to have at least 128 participants in our study.

### Participants

One hundred and seventy-three undergraduate female students from France (N = 89) and Belgium (N = 84) took part in the study (*M*_age_ = 19.66 years, SD = 2.04). All participants were fluent French speakers and completed the study in French. As we had limited access to participants in each of the locations, the same study was conducted in Belgium and in France to increase sample size. Instructions and recruitment strategy were the same for both studies. In both locations, participants voluntarily applied to take part in the study, which represented one choice among several other eligible studies, and received course credit as compensation for their participation. The study was presented as a study on “advertisement perception”. The mean body mass index was within the normal weight range in the sample (*M* = 21.27, *SD* = 4.41), with 65.3% of participants of normal weight (18.5 ≤ BMI ≤ 25), 23.1% underweight (18.5 < BMI), 9.3% overweight (25 < BMI ≤ 30) and 2.3% obese (BMI > 30). A t-test revealed that samples did not differ in BMI, *t*(169) = 1.162, *p* = .25. Demographic information for each sample are available in Table 1.

**Table 1:** Descriptive statistics per sample.

	Mean Age (SD)	Mean BMI (SD)
France *(N* = 88)	18.48 (1.15)	21.64 (3.96)
Belgium *(N* = 83)	20.90 (2.05)	20.85 (4.87)

Participants were informed about the study and gave their consent before participating. The ethical committee of the Psychological Science Research Institute, Université Catholique de Louvain, approved the study protocol.

### Procedure and materials

The experiment was conducted on a computer using Psychopy® software [39]. All materials and instructions were displayed in French.

#### Body dissatisfaction

Body dissatisfaction was measured with the Body Dissatisfaction subscale of the Eating Disorder Inventory [40]. Participants indicated their agreement/disagreement with nine statements (e.g., “I think that my stomach is just the right size”) using 7-point Likert scales (1 = completely disagree, 7 = completely agree). This construct was assessed to test whether thin-ideal exposure and cognitive load affect negative thought accessibility when body dissatisfaction is controlled for.

#### Cognitive load manipulation

Digit retention instructions, adapted from Gilbert et al. [25], were used to manipulate cognitive load. Participants were randomly allocated either to a low or to a high cognitive load condition, in a between-participants design. Participants in the low load condition had to retain four digits (2-9-5-10), whereas participants in high load condition had to retain ten digits (8-4-3-9-5-2-10-1-7-6). The digits were displayed on the computer screen until participants declared that they had memorized them. Participants were then informed that they would have to recall the digits at the end of the procedure.

#### Thin-ideal exposure manipulation

Participants were exposed either to thin-ideal pictures or to pictures related to feminine beauty. Participants in the experimental condition were exposed to three thin-ideal images in a random order. These three pictures of women in underwear had been altered in Photoshop® in order to make the models look thinner. They were taken from Chatard and Selimbegovic [6]. Participants in the control condition, were exposed to three pictures of feminine fashion items: a handbag, a lipstick, and a scarf (Appendix 1). They were instructed to observe the different pictures with attention.

#### Thoughts accessibility measure

All participants then completed a lexical decision task in which they had to indicate whether each of the letter strings successively presented on the computer screen formed a word or not by pressing the corresponding keys on the keyboard. Among the words, twenty were neutral, twenty were positive, and twenty were negative. There were sixty non-words that corresponded to the words with interchanged letters.

At the end, participants completed a socio-demographic questionnaire in which they reported their age, their native language, the numbers they retained, their height, and their weight. They were then thanked and debriefed.

## Results

### Data preparation

#### Thought accessibility

Incorrect responses were excluded from the analysis (5.61% for negative and 7.92% for positive words), and latencies greater than 2000 ms were replaced by 2000 ms [41]. Reduced mean latencies for a category of words indicated greater cognitive accessibility of the related content. Latencies to non-word stimuli were not analyzed.

### Preliminary analyses

#### Body dissatisfaction

Body dissatisfaction was unrelated to both negative and positive thought accessibility, *r*(173) = −.07, *p* = .40, and *r*(173) = −.04, *p* = .62, respectively. Moreover, when body dissatisfaction scores were controlled for, the significance of the results reported below did not change. Thus its effect is not discussed further.

### Main analyses

To test our main hypothesis, we conducted a 2 (thin-ideal exposure: thin ideal vs. fashion items) x 2 (cognitive load: low vs. high) ANCOVA on latencies to negative and positive words, with latencies to neutral words as a covariate.

#### Negative thought accessibility

Two participants were excluded because they were detected as outliers on latencies to both negative and neutral words (z scores > 3).

Latencies to neutral words significantly predicted latencies to negative words, *F*(1, 166) = 605.11, *p* < .0001 , *η*^2^_p_ = .79. Over and above this effect, the main effects of thin-ideal exposure, *F*(1, 166) = 0.59, *p* = .45, *η*^2^_p_ = .004, and cognitive load, *F*(1, 166) = 1.16, *p* = .28, *η*^2^_p_ = .007, were not significant. However, the interaction effect was significant, *F*(1,166) = 6.35, *p* = .013, *η*^2^_p_ = .04. To understand this interaction, we tested the effects of thin-ideal exposure on each level of cognitive load. Women under high load showed lower mean latencies to negative words in the thin-ideal condition than in the fashion accessories condition, *t*(166) = − 2.30, *p* = .02. In contrast, the effect of thin-ideal exposure was not significant for women under low load, *t*(166) = 1.28, *p* = .20. Means and standard deviations are presented in Table 2.

**Table 2:** Mean recognition latencies (in milliseconds) for positive and negative words as a function of exposure condition and cognitive load (adjusted and unadjusted for neutral words).

	Latencies for positive words	
	Unadjusted means	
	Low load *M (SD)*	High load *M (SD)*
Thin ideal	682 (122)	697 (140)
Fashion accessories	708 (105)	657 (107)
	Adjusted means	
	Low load *M (SE)*	High load *M (SE)*
Thin ideal	688 (8)	692 (8)
Fashion accessories	679 (8)	688 (9)
	Latencies to negative words	
Exposure	Unadjusted means	
	Low load *M (SD)*	High load *M (SD)*
Thin ideal	714 (125)	711 (129)
Fashion accessories	733 (126)	705 (110)
	Adjusted means	
	Low load *M (SE)*	High load *M (SE)*
Thin ideal	720 (9)	706 (9)
Fashion accessories	704 (9)	736 (10)

#### Positive thought accessibility

Three participants were excluded because they were detected as outliers on latencies to positive, or both positive and neutral words (z scores > 3).

Latencies to neutral words significantly predicted latencies to positive words, *F*(1, 165) = 618.58, *p* < .0001 , *η*^2^_p_ = .79. The main effects of thin-ideal exposure, *F*(1, 165) = 0.65, *p* = .42, *η*^2^_p_ = .004, and cognitive load, *F*(1, 165) = 0.57, *p* = .45 , *η*^2^_p_ = .003, were not significant, and neither was the interaction of exposure and load, *F*(1,165) = 0.07, *p* = .79, *η*^2^_p_ < .001. Thus, positive thought accessibility remained unaffected by the experimental manipulations. Means and standard deviations are presented in Table 2.

### Complementary analyses

#### Country effects

As the study was simultaneously conducted in France and in Belgium, we ran analyses to test whether the country factor qualified the effects of thin-ideal exposure and cognitive load on negative thought accessibility. We thus repeated the reported analyses, with country as an additional factor, on both negative and positive thought accessibility (latencies to neutral words were covaried). For *negative thoughts*, there was an interaction of exposure and country, *F*(1,162) = 7.37, *p* = .007, *η*^2^_p_ = .003, showing that irrespective of cognitive load, exposure to the thin ideal increased negative thought accessibility in France, *t*(162) = −2.46, *p* = .015, but not in Belgium, *t*(162) = 1.40, *p* = .16. Even with this interaction controlled for, the interaction of exposure and cognitive load was still significant, *F*(1,162) = 7.07, *p* = .009, *η*^2^_p_ = .04, and was not qualified by a three-way interaction with country, *F*(1,162) = 0.15, *p* = .70, *η*^2^_p_ = .001. For *positive thoughts*, none of the effects involving the country factor were significant, and controlling for the country factor did not change the significance of the results reported above.

## Discussion

The present study aimed to test whether negative consequences after thin-ideal exposure ensued from a resource-independent, automatic process. The results indicated that high cognitive load facilitated an increase in negative thought accessibility after thin-ideal exposure. This lends support to the hypothesis of an underlying resource-independent process. To the extent that negative effects of thin-ideal exposure are most often accounted for by social comparison processes [5, 3, 24, 29], this study provides a theoretical contribution to the literature by suggesting that social comparison is an automatic process, from the viewpoint of resource independence (i.e., efficiency). Indeed, Moors and De Houwer [42] argued that if an outcome is observed on an implicit measure, it means that process underlying this outcome is automatic. Thus, based on their assumption and previous research that suggested that thin-ideal exposure involves social comparison process [5, 3, 24, 29], it is reasonable to conclude that social comparison could be an efficient process requiring few cognitive resources. The present work thus complements recent findings reported by Chatard et al. [22], who demonstrated that subliminally presented thin ideal pictures produced an increase in body-focused anxiety, and also argued in favor of the hypothesis of automatic social comparison (from the viewpoint of consciousness).

These results are consistent with some previous research [22, 25, 26] but stand in contrast to Want et al.’s [23, 33]. As argued above, and in line with Want et al.’s [23] suggestion, this apparent inconsistency might be explained by the fact that they used explicit dependent measures, while we relied on an implicitly measured outcome. Indeed, given that it has become normative for women to be unsatisfied with their physical appearance and to consider themselves “too fat” [28], it is not unlikely that demand characteristics may contribute to thin-ideal exposure effects on explicit measures. This is especially likely when the study design relies on repeated outcome measures (as in Want et al.’s studies). In such contexts, participants typically evaluate their own appearance (and/or mood) before being exposed to a standard of beauty, and they complete the same measures again after exposure. It is thus likely that the procedure is to a certain degree transparent to participants. Accordingly, it is possible that cognitively occupied participants failed to respond in line with demand characteristics, which could thus have significantly attenuated the pre-post difference in the low load condition in Want et al.’s studies. In contrast, the current study not use a repeated-measures design, and we relied on a reaction time-based measure. Such a procedure makes it highly unlikely for word recognition latencies to be affected by demand characteristics. In contrast to Want et al.’s [23, 33], we did not observe any effect when cognitive load was relatively low, but we observed a negative effect of thin-ideal exposure when participants were cognitively occupied.

It remains to elucidate why there was no effect of thin-ideal exposure in the low load condition, assuming that the underlying process is indeed efficient. This result was actually unexpected. However, given that more resources were available in the low load than in the high load condition, it is possible that controlled processes could have interfered with reactions to thin-ideal exposure in the low load more than in the high load condition. For instance, Gilbert et al. [39, 25] correction model of social comparison suggests that people engage in social comparison automatically and spontaneously. However, when their resources are not taxed, they can *subsequently* correct their judgment, for instance, by taking into account the (non-)diagnosticity of the comparison target. In contrast, when participants are cognitively occupied, this costly correction that allows them to reverse or suppress the effects of the comparison is no more possible. Gilbert et al. [25] argued that if the effect appears under high cognitive load but not under low load, as is the case here, this constitutes a proof of correction processes.

A strength of the reported findings is that they were not qualified by an interaction with the country in which the data were collected. Although some differences between the French and the Belgian participants appeared, the cognitive load by thin-ideal exposure interaction was unaffected by country. This suggests that our findings are generalizable across at least two countries. Thus, it reveals a certain robustness of the phenomenon under study.

### Limitations and future research

Despite the strengths of the present findings, certain limitations have to be acknowledged. In the control condition, women wereexposed to fashion accessories and not to body shapes. Although such a control condition reflects the type of content that can be found in fashion magazines along with pictures of the thin ideal, it does not allow attributing the reported effects specifically to thinness. On the positive side, it does provide the opportunity to compare thin-ideal exposure to a “baseline” condition that does not focus participants on body shape and thus probably more closely reflects their spontaneous thoughts. This control condition prevents the body shape comparison that occurs in the thin-ideal condition and thus supports the conclusion that the effect is indeed driven by social comparison.

In addition, as underlined by Want et al. [23], we cannot ascertain that the cognitive load manipulation used in the present study parallels the mental preoccupation with which women are confronted in their daily lives. Future research could rely on a different cognitive load manipulation that more closely resembles everyday life conditions. Moreover, a different control condition might be used that could pinpoint comparisons with *thin* models (and not average-size good-looking models) as the causal factor underlying the negative effects of thin-ideal exposure. Ideally, the only thing that should be different between the experimental and the control condition is the thinness of the model. A final limitation pertains to the completion of the body dissatisfaction scale prior to the experimental manipulation. Explicit questions about body-related feelings could prime women to focus on their own body before the exposure to thin-ideal pictures, thereby facilitating the occurrence of thin-ideal exposure effects. Thus, it remains to be seen whether the same pattern of results would be observed if women are not led to evaluate their body satisfaction at the beginning of the procedure. Furthermore, from the viewpoint of external validity, in everyday life, women may not always be focused on their bodies before being exposed to thin-ideal pictures in advertisement. Future research might experimentally manipulate the positioning of the body dissatisfaction scale in the experimental procedure to study the importance of body focus in the effects reported in the present paper.

## Conclusion

Despite the limitations acknowledged above, the present findings complement the extant literature and revitalize the open question about social comparisons as automatic processes. These findings suggest that thin-ideal exposure may provoke an automatic increase of negative thought accessibility. Assuming that thin-ideal exposure induces social comparison, these results suggest that social comparison could be an efficient process, requiring few cognitive resources. If women automatically compare themselves to models who represent the thin ideal, this might explain why some strategies developed to prevent implicit negative effects of such comparisons are inefficient (e.g., disclaimers on airbrushed photos; see [43]). Indeed, such strategies specifically focus on controlled processing. This further suggests that prevention strategies focused on implicit processes may be more efficient. Although further research is necessary to replicate and extend the present results, they significantly contribute to a deeper understanding of the processes behind negative effects of thin-ideal exposure. Somewhat paradoxically, the present findings also suggest that it is precisely when women do not pay much attention to the thin ideal (because they are cognitively busy) that they suffer the most from this unattainable standard of comparison.

## Supporting information

S1 Appendix This is the appendix 1. (DOCX)
