# Supplementary figures and images for: Automatic social comparison: Cognitive load facilitates an increase in negative thought accessibility after thin ideal exposure among women
Source: PLoS One. 2018 Mar 28;13(3):e0193200. doi: 10.1371/journal.pone.0193200 (PMC5873941; doi:10.1371/journal.pone.0193200)

Appendix A

Thin-ideal pictures


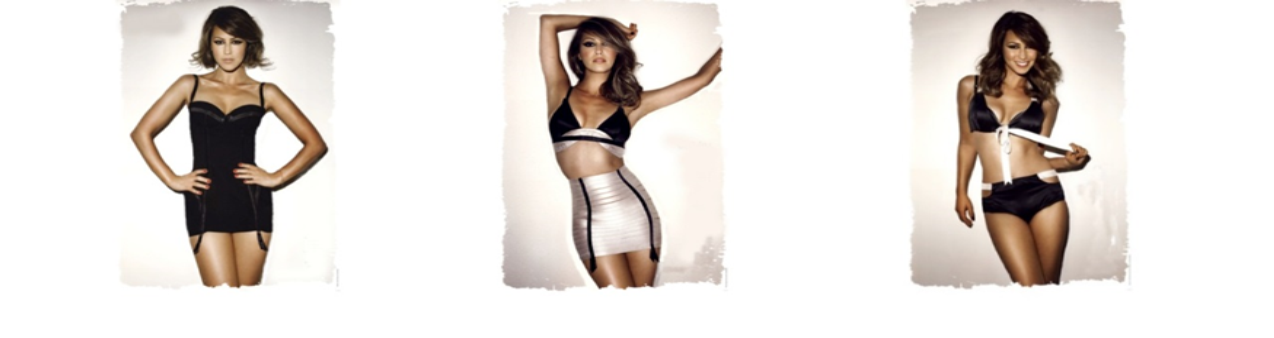


Fashion accessories pictures


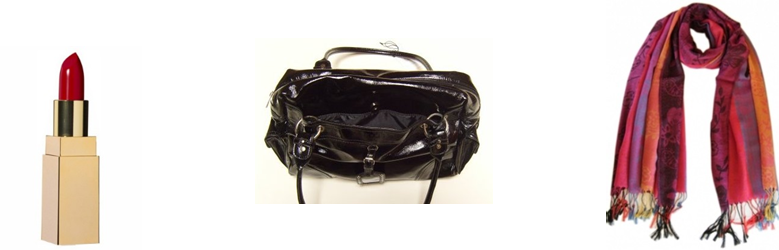

Supplement: S1 Appendix — (DOCX) [file pone.0193200.s001.docx]
